# The effect of newly designed dual-channel elastomeric pump for intravenous patient-controlled analgesia after total laparoscopic hysterectomy: a randomized, double-blind, prospective study

**DOI:** 10.1186/s13741-022-00282-z

**Published:** 2022-10-12

**Authors:** Seok Kyeong Oh, Heezoo Kim, Young Sung Kim, Chung Hun Lee, Jung Suk Oh, Dae Hui Kwon

**Affiliations:** 1 Department of Anaesthesiology and Pain Medicine, Korea University Guro Hospital, Korea University College of Medicine, Seoul, Republic of Korea; 2 Department of Obstetrics and Gynecology, Bucheon Sejong Hospital, Bucheon, Republic of Korea

**Keywords:** Patient-controlled analgesia, Postoperative nausea and vomiting, Hysterectomy, Patient satisfaction, Quality of recovery

## Abstract

**Background:**

A newly designed intravenous patient-controlled analgesia (PCA) device with a dual-channel elastomeric infusion pump has been recently introduced. One channel is a continuous line with a constant flow rate basal infusion, while the other channel has an adjustable flow rate and bolus function and is labeled as a selector-bolus channel. This study compared dual and single-channel intravenous PCA in terms of clinical effect and quality of recovery.

**Methods:**

Eighty-four patients undergoing total laparoscopic hysterectomy were randomly allocated to a 1-channel group (*n* = 41) or a 2-channel group (*n* = 43). Only the selector-bolus channel was utilized, but the continuous channel was not utilized in the 1-channel group, but both channels were utilized in the 2-channel group. In the 1-channel group, 16 μg/kg of fentanyl, 2 mg/kg of ketorolac, and 12 mg of ondansetron with normal saline were administered to the selector-bolus channel and normal saline only in the continuous channel for blinding. In the 2-channel group, 16 μg/kg of fentanyl was administered to the selector-bolus channel, and ketorolac (2 mg/kg) and ondansetron (12 mg) were administered via the continuous channel.

The quality of recovery was evaluated preoperatively and 24 h postoperatively using the Quality of Recovery-40 (QoR-40). Cumulative PCA consumption, postoperative pain rated using the numeric rating scale (NRS; during rest/cough), and postoperative nausea were evaluated 6, 12, 24, 36, and 48 h after surgery. Incidence of vomiting and use of antiemetics and rescue analgesics was measured.

**Results:**

The 24-h postoperative QoR-40 score was higher in the 2-channel group than in the 1-channel group (*P*=0.031). The incidence of nausea at 12 h and 36 h was significantly higher in the 1-channel group (*P*=0.043 and 0.040, respectively), and antiemetic use was more frequent in the 1-channel group (*P*=0.049). Patient satisfaction was higher in the 2-channel group (*P*=0.036). No significant differences were observed in pain scores during resting/cough or cumulative PCA consumption.

**Conclusions:**

The 2-channel PCA showed better patient satisfaction with higher QoR-40 during the recovery compared with the 1-channel PCA. Better satisfaction was associated with lower nausea and reduced rescue antiemetics by maintaining the infusion of adjuvant analgesic agents and antiemetic agents constantly by utilizing dual channels.

**Trial registration:**

Registered at ClinicalTrials.gov, NCT04082039 on 9 September 2019.

## Background

Intravenous patient-controlled analgesia (PCA) is commonly used to relieve postoperative pain, and opioids are the most commonly used drugs for intravenous PCA (Grass 2005; Macintyre 2001; Oh et al. 2019; Rawal 2016). However, opioids cause nausea, vomiting, sedation, and respiratory depression as side effects; thus, non-opioid analgesics such as ketorolac or nefopam were added as adjuvants to provide an opioid-sparing effect (Chou et al. 2016; Koh et al. 2015). Additionally, antiemetic agents, such as ondansetron or ramosetron, can be administered together in a pump (Koh et al. 2015; Lee et al. 2011; Reddy et al. 2019). When severe opioid-related side effects occur, PCA administration is suspended. In this situation, both the analgesic effect of the opioid and the effect of adjuvant agents cannot be applied because they are administered together in a single chamber.

**Fig. 1:**
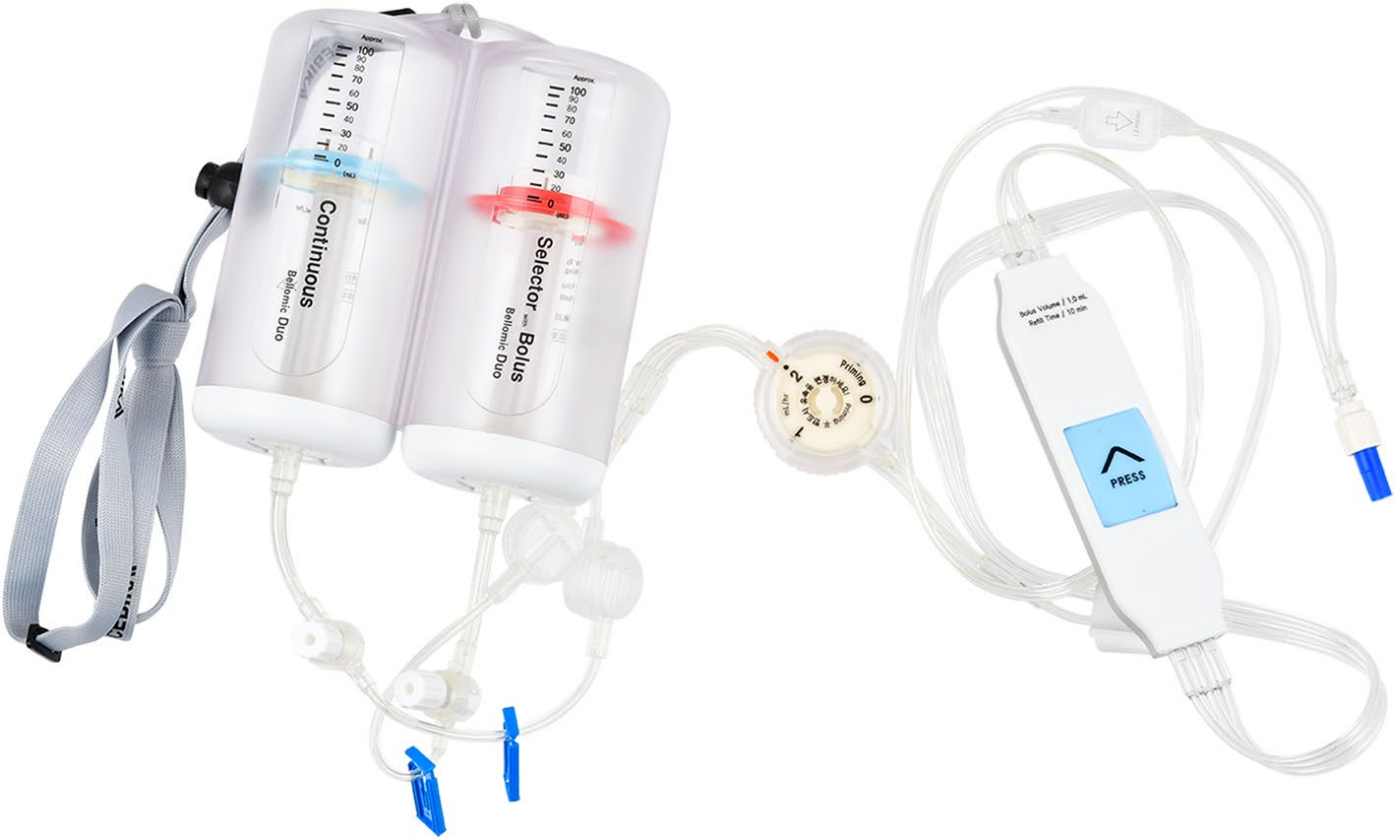
Photograph of the Bellomic Duo® silicone balloon infuser

The Bellomic Duo® silicone balloon infuser, a dual continuous petite type (Cebika, Uiwang-si, Gyeonggi-do, Republic of Korea) is a newly designed PCA device that has a dual infusion elastomeric pump with two balloon chambers. One channel is a continuous line with a constant flow rate basal infusion, while the other channel has an adjustable flow rate and bolus function, and is labeled as a selector-bolus channel (Fig. 1).

Conventional elastomeric PCA devices are used to administer a mixture of drugs from one chamber (Shin et al. 2014); therefore, the administration of all the drugs is halted by clipping. Additionally, mixing different drugs in the same syringe or infusions is not generally recommended because of the incompatibility of mixtures including precipitation, ionic reactions, and evolution of gas (Murney 2008). In contrast, this newly designed device can be controlled as needed by administering adjuvant analgesics or antiemetic agents through another chamber and line. It will be expected to facilitate patient management and increase patient satisfaction and recovery.

Recently, with advances in perioperative care, overall assessments of the quality of life of patients undergoing surgery provide more appropriate measures of the effects of anesthetic or surgical care on patients’ recovery and their levels of satisfaction rather than the quantitative assessment of fragmentary postoperative indicators, such as morbidity or mortality rates (Choi et al. 2017). Quality of recovery after anesthesia is a significant indicator of the early postoperative health status of patients (Myles et al. 2000). The quality of the recovery-40 (QoR-40) questionnaire is a widely used, self-rated, self-completed, and reliable multi-dimensional assessment tool for postoperative patients. The Korean version of the QoR-40 questionnaire was developed and found to be as acceptable and reliable as the original English QoR-40 for Korean patients after surgery (Lee et al. 2018).

This study was performed to compare dual-channel intravenous PCA with single-channel IV-PCA in terms of their clinical effect, including pain scores and opioid consumption, adverse effects, overall satisfaction, and quality of recovery as assessed using the QoR-40 questionnaire, in patients undergoing total laparoscopic hysterectomy (TLH). We hypothesized that the dual-channel PCA could provide comparable analgesic efficacy with higher satisfaction and quality of recovery than single-channel PCA.

## Methods

### Patients and study design

This study was approved by the Ethical Committee of the Korea University Guro Hospital Institutional Review Board, Seoul, Republic of Korea (ref.: 2019GR0294) on 21 August, 2019, and registered at the ClinicalTrials.gov (ref.: NCT04082039). Between September 2019 and June 2020, female patients aged 19–75 years with American Society of Anesthesiologists (ASA) physical status I–II, who were scheduled to undergo elective TLH under general anesthesia, were enrolled in this study after providing written informed consent. Patients were excluded if they had a body mass index >30.0 kg/m^2^, known hypersensitivity to the drugs used in this study, significant liver or renal dysfunction, or a history of drug abuse or dependence, recent major procedure, or surgery.

### Randomization and allocation

Participants were randomly assigned to either a 1-channel or a 2-channel group using a web-based computer-generated list and were unaware of their assignment. The randomized numbers were kept by a non-blinded researcher (SKO) in sequentially numbered envelopes, and the envelope was opened in the operating room only by the non-blinded anaesthesiologist (SKO) who was responsible for setting the PCA pump as per the study protocol. Other investigators who provided anesthesia and assessed the study endpoints after the operation in the post-anesthesia care unit (PACU) and ward were blinded to the group assignment. All PCA devices were applied to patients with labels only denoting the patient’s study number, so that neither the patient nor medical care providers and investigators could recognize the allocation.

### Anesthesia

Following the patient’s arrival in the operating room, routine physiological monitoring, such as pulse oximetry, electrocardiography, and noninvasive arterial blood pressure measurements, were performed. The bispectral index (BIS VISTA™; Aspect Medical Systems Inc., Norwood, MA, USA) was used to monitor the depth of hypnosis and maintained between 40 and 60 during surgery.

For preemptive pain management and maintenance of hemodynamic stability during the initial phase of the operation, 50 μg of fentanyl was administered intravenously in both groups immediately before the induction of anesthesia. Anesthesia was induced using 2 mg/kg of intravenous propofol, followed by 0.6 mg/kg rocuronium, which was administered after the loss of consciousness (BIS < 60) for endotracheal intubation and maintained with desflurane and 50% oxygen in the air. The concentration (vol%) of desflurane was titrated using the bispectral index value. The esophageal temperature was maintained at approximately 36°C using a warm air blower. Five minutes after the uterus incision, 50 μg of fentanyl was injected to control the pain from surgical stimuli perioperatively. An addition, 50 μg of fentanyl was administered 30 min after the last dose of fentanyl until the laparoscopic procedure ended. At the end of the surgical procedure, neuromuscular blockade was reversed using 0.4 mg glycopyrrolate and 10 mg pyridostigmine. After confirming the response to verbal commands and spontaneous respiration, tracheal extubation was performed. If the mean arterial pressure decreased or increased by more than 30% from baseline, 4 mg of ephedrine or 0.5 mg of nicardipine, respectively, were administered.

### PCA protocol

The Bellomic Duo® silicone balloon infuser has a dual-channel infusion elastomeric pump. The continuous channel is a line with a fixed flow rate of 2 mL/h for basal infusion, and the selector-bolus channel can be set to 0, 1, and 2 mL/h with an adjustable flow rate and bolus function. The bolus volume is 1 mL, and the lock-out period is 10 min. The volume of each chamber was 100 mL, and the total volume was 200 mL. In this study, the Bellomic Duo® was used for both groups, but the same regimen of drugs was administered by dividing it into different channels. The methods were as follows for each group.

In the 1-channel group, fentanyl 16 μg/kg, ketorolac 2 mg/kg, and ondansetron 12 mg with normal saline to reach a total volume of 100 mL were administered in the selector-bolus channel at a rate of 2 mL/h, and 100 mL of normal saline only was present in the continuous channel to blind the group allocation. Therefore, only the selector-bolus channel was utilized, but the continuous channel was not utilized in the 1-channel group.

Meanwhile, both channels were utilized in the 2-channel group. In the 2-channel group, fentanyl 16 μg/kg with normal saline to a total volume of 100 mL was administered via the selector-bolus channel at a rate of 2 mL/h, and ketorolac 2 mg/kg, ondansetron 12 mg with normal saline to a total volume of 100 mL was administered via the continuous channel.

**Fig. 2:**
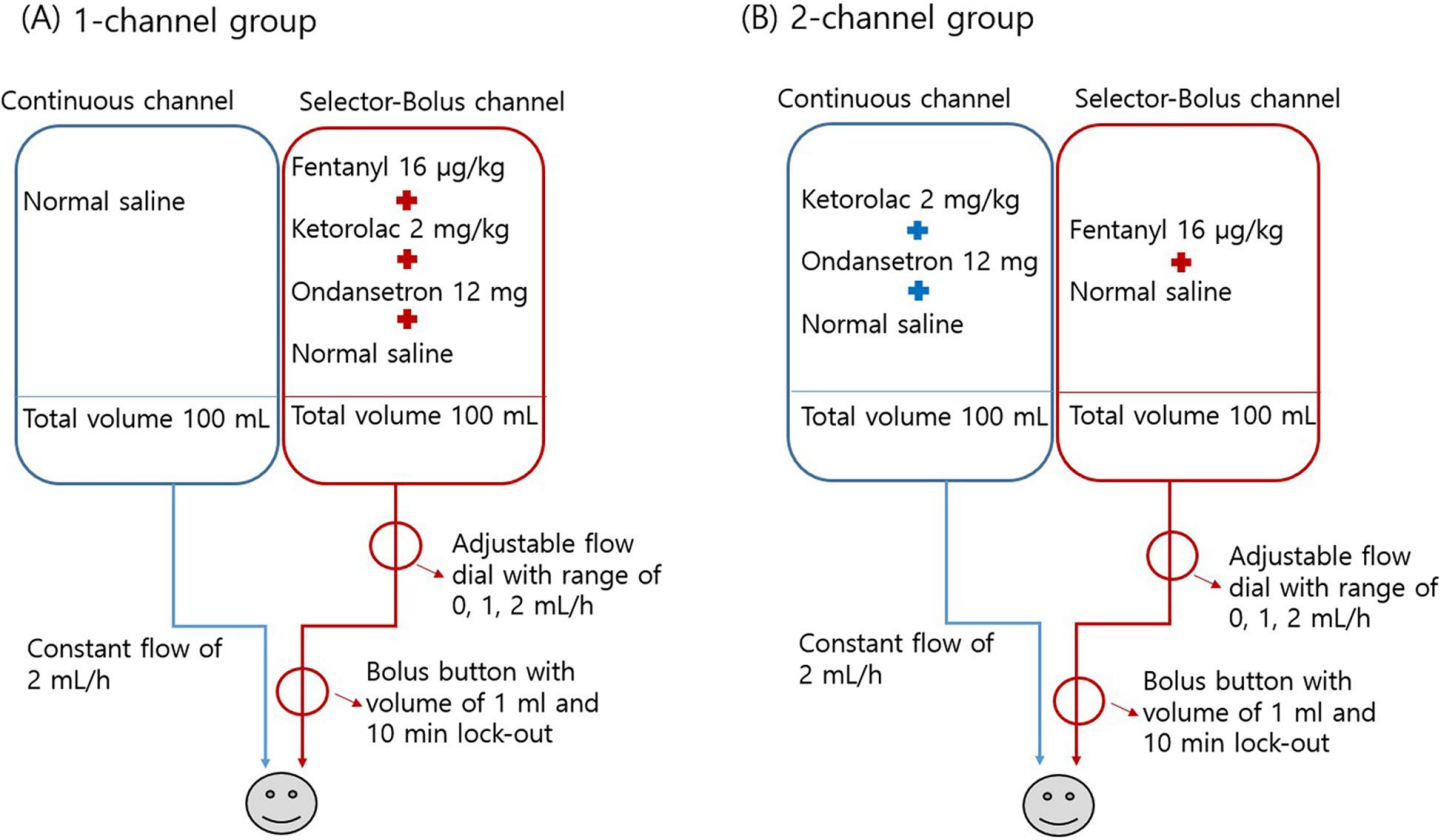
Schematic of the PCA device and its application. **A** In the 1-channel group, only the selector-bolus channel was actually utilized. **B** In the 2-channel group, both the selector-bolus channel and continuous channel were utilized by dividing the drugs between the two. The adjustable flow dial has a range of 0, 1, and 2 mL/h, but only 0 or 2 mL/h were used in both groups per our study protocol

A schematic drawing of the PCA device and its application is shown in Fig. 2.

### Postoperative management and assessment

In the PACU, the level of the patient’s pain was assessed using a verbal pain score (VPS; 0–3; 0 = no pain, 1 = mild pain, 2 = moderate pain, 3 = intense pain) (Du Manoir et al. 2003) at 10-min intervals, and the sedation score (0–3; 0 = clearly conscious; 1 = temporarily drowsy; 2 = drowsy but responsive to verbal communication; and 3 = drowsy without response to verbal communication) (Du Manoir et al. 2003) was also evaluated every 10 min. Twenty-five micrograms of fentanyl was administered if the patient had a VPS ≥ 2, respiratory rate ≥ 10 per minute, SpO_2_ ≥ 95%, and sedation score ≤ 1. When the VPS and sedation score were ≤ 1, the PCA device was connected.

After transferring the patients to the ward, the pain was assessed using a numeric rating scale (NRS; 0–100; no pain [0] to worst pain imaginable [100]) at 6, 12, 24, 36, and 48 h after surgery. The cumulative consumption of PCA over 48 h and the occurrence of adverse effects, such as nausea and vomiting, dry mouth, dizziness, urinary retention, headache, sedation, itchiness, shivering, respiratory depression, confusion, hypotension, and bradycardia, were monitored. Respiratory depression was defined as a respiratory rate < 10 per min or oxygen saturation < 90% for >1 min (Tsui et al. 1997). The patient’s overall satisfaction score (0–10; from unsatisfied [0] to fully satisfied [10]) was also assessed. If the postoperative pain control in the ward was insufficient (NRS score for pain > 40) or if an additional analgesic was requested by a patient, paracetamol 1 g or ketorolac 30 mg was planned as a rescue analgesic.

Nausea was assessed (0 = none, 1 = mild, 2 = moderate, and 3 = severe), and 4 mg of ondansetron was planned as a rescue antiemetic agent if a patient suffered severe nausea or retching/vomiting (Choi et al. 2017).

In both groups, the flow rate of the selector-bolus channel was initially set at 2 mL/h. When opioid-related side effects such as nausea, vomiting, or dizziness occurred, infusion of the selector-bolus channel was suspended by adjusting the flow rate to 0, while the continuous channel remained infused. After the symptoms were relieved and/or pain control was demanded by patients, the selector-bolus channel was re-infused at a rate of 2 mL/h and re-suspended in the recurrence of unbearable side effects. The decision of rate change was made by the evaluating doctors who round the patients at 6, 12, 24, 36, and 48 h after surgery and depending on the case the nurse in the ward gives a notice.

Postoperative quality of recovery was evaluated by the QoR-40, which has 40 questions in five domains (Choi et al. 2017). Each question that assesses patient recovery uses a five-point Likert scale (1 = none of the time, 2 = some of the time, 3 = usually, 4 = most of the time, and 5 = all of the time). The five domains are physical comfort, pain, physical independence, psychological support, and emotional state. The global QoR-40 scores range from 40 to 200. All patients answered the questionnaire on the day before surgery and 24 h after surgery.

### Evaluation of outcomes

The outcomes measured included (1) QoR-40 score on the day before surgery and at postoperative 24 h (primary endpoint), (2) VPS (0–3) measured every 10 min at the PACU, (3) the sedation score (0–3) measured every 10 min at the PACU, (4) rescue fentanyl administration in the PACU, (5) NRS score (0–100) at rest and while coughing in the ward, (6) cumulative PCA consumption over 48 h (mL), (7) nausea and vomiting (0–3) for 48 h after PCA connection, (8) occurrence of other side effects in the PACU and in the ward for 48 h after PCA connection, (9) incidence of the selector-bolus channel at a rate of 0 mL/h due to severe side effects, (10) patient’s overall satisfaction score (0–10; 0 = unsatisfied to 10 = fully satisfied), and (11) length of postoperative hospital stay.

### Statistical analysis

The primary endpoint was the QoR-40 score at 24 h postoperatively. The sample size calculation was based on a mean QoR-40 score of 167 ± 23 at 24 h after surgery, which was derived from a previous study (Myles et al. 2000). We considered that a difference of more than 10% between groups with respect to the QoR-40 score was clinically significant following a previous study (Choi et al. 2017). Based on the assumption that the allocation ratio of the two groups was 1, 40 subjects were required in each group to achieve an alpha value of 0.05 and power of 80%. We aimed to assign 45 patients to each group after accounting for a 10% dropout rate.

Statistical analyses were performed using SPSS software (version 20.0; IBM, Corporation, Armonk, NY). The normal distribution of continuous data was first evaluated using the Shapiro–Wilk test (*P* > 0.05). Normally distributed data were analyzed using Student’s *t* test, and non-normally distributed data were analyzed using the Mann–Whitney *U* test. Student’s *t* test was used to compare the age, height, weight, anesthesia time, and operation time, while the Mann–Whitney *U* test was used for the QoR-40 score, rescue fentanyl dose, and PCA connection time at the PACU.

Ordinal parameters, including the VPS and sedation score at the PACU and patient’s overall satisfaction score, were compared using the Mann–Whitney *U* test, while categorical variables, including the ASA classification and incidence of adverse events, were compared using a chi-squared test or Fisher’s exact test. The change in the QoR-40 score from the preoperative period to the postoperative 24 h in each group was analyzed using the Wilcoxon signed-rank test, and the changes over time in the NRS score at rest and while coughing at 6, 12, 24, 36, and 48 h after surgery, and cumulative PCA consumption in the ward were compared using repeated-measures analysis of variance.

Data are expressed as the mean ± SD, median [IQR], or number of patients (%). *P* values were two-tailed, and a *P* value of less than 0.05 was considered statistically significant.

## Results

**Fig. 3:**
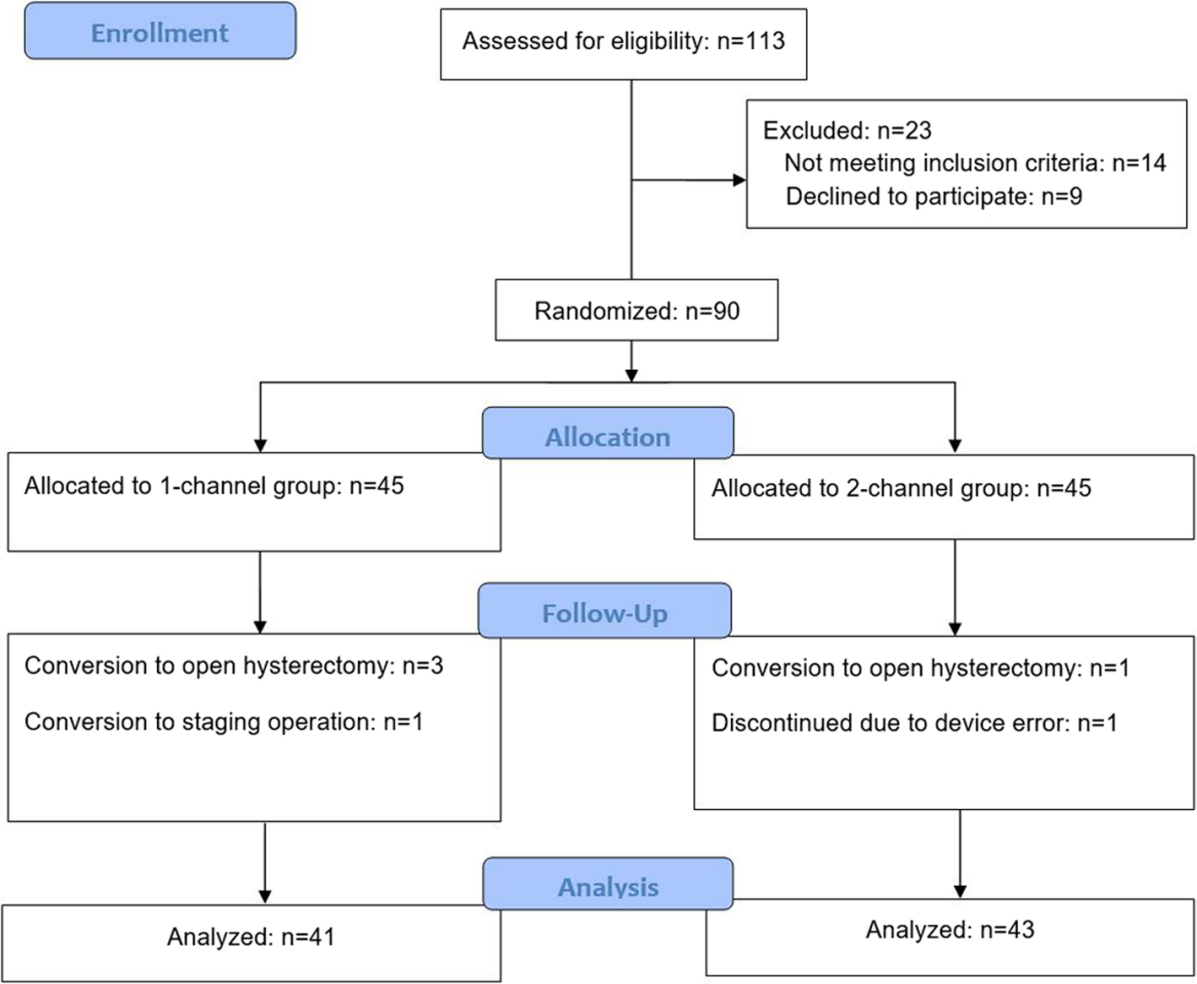
A flowchart describing patient recruitment, randomization, and withdrawal

A total of 113 patients were assessed for eligibility, and 23 patients were excluded for noncompliance with the study protocol; therefore, 90 patients (45 for each group) were randomized. Among these, four operations were converted to open hysterectomy, one to a staging operation, and one patient discontinued due to device error (underdosed infusion of the selector-bolus line channel). Finally, 41 patients in the 1-channel group and 43 in the 2-channel group were analyzed (Fig. 3). There was no significant intergroup difference in baseline patient characteristics, operation types, and operation and anesthesia times (Table 1).

**Table 1:** Demographic and clinical data

	1-channel (*n*=41)	2-channel (*n*=43)	*P* value
Age, year	49 [43–54]	49 [47–56]	0.407
ASA, I/II	7/34	6/37	0.768
Height, m	1.57 ± 0.06	1.60 ± 0.06	0.055
Weight, kg	58.8 [51.4–69.0]	59.0 [56.0–65.0]	0.816
BMI, kg/m^2^	25.2 ± 4.3	23.9 ± 3.2	0.144
Operation type			0.780
TLH only	32	31	
TLH with lymphadenectomy	2	2	
TLH with salpingo-oophorectomy or salpingectomy	7	10	
Operation time, min	70 [44–98]	63 [44.5–86]	0.177
Anaesthesia time, min	115 [75–135]	105 [82–125]	0.152
Intraoperative fentanyl, μg	100 [100-150]	100 [100-150]	0.702

Values are mean ± SD, median [IQR], or number of patients

All the subjects included in this study were female

Abbreviations: *ASA* American Society of Anesthesiologists physical status classification, *BMI* body mass index, *TLH* total laparoscopic hysterectomy

**Fig. 4:**
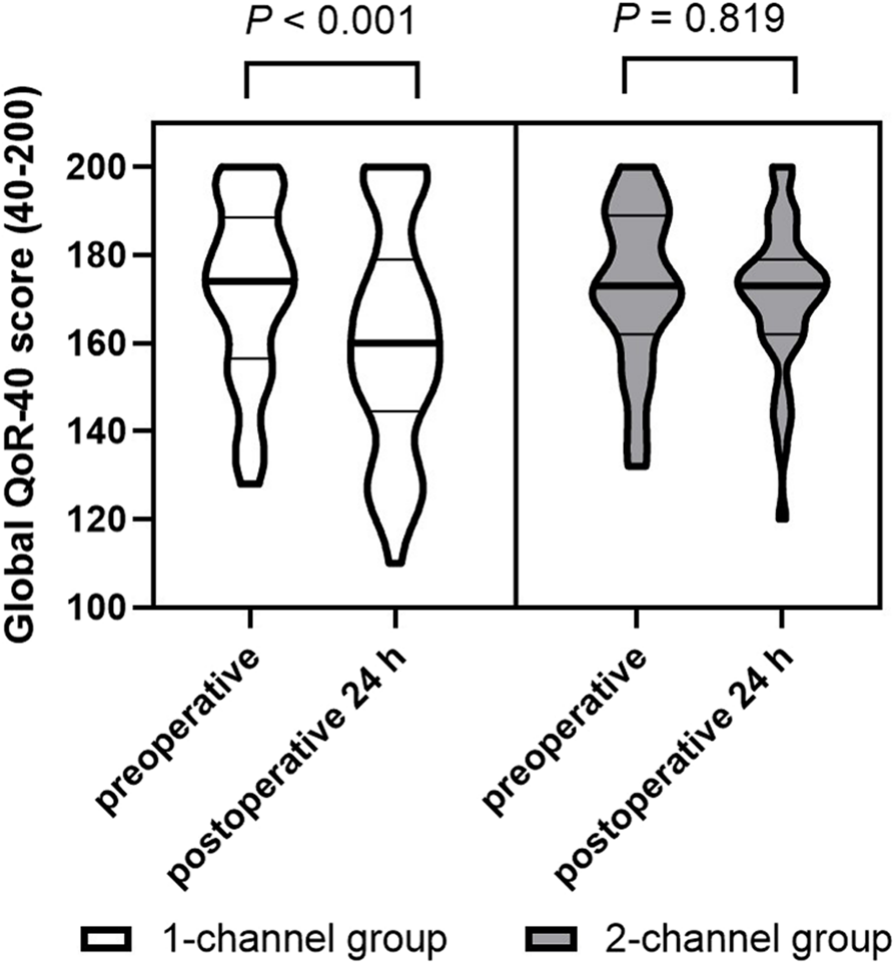
Violin plots of the global QoR-40 at preoperative period and preoperative 24 h. The change in the QoR-40 score from the preoperative period to the postoperative 24 h in the 1-channel group was significantly decreased, but not in the 2-channel group (*P* < 0.001 and *P* = 0.819, respectively, by the Wilcoxon signed-rank test). Abbreviations: QoR-40, quality of recovery-40 questionnaire

The preoperative QoR-40 score was comparable between the two groups, but the postoperative 24 h QoR-40 score was higher in the 2-channel group than in the 1-channel group (171 [162–175] vs. 157 [142–182.5]; *P* = 0.048) (Table 2). The change in the QoR-40 score from the preoperative period to the postoperative 24 h in the 1-channel group was significantly decreased, but there was no significant change in the 2-channel group (*P* < 0.001 and *P* = 0.819, respectively) (Fig. 4).

**Table 2:** Postoperative outcomes

	1-channel (*n*=41)	2-channel (*n*=43)	*P* value
Global QoR-40 score			
Preoperative	172 [153–192]	172 [157–189]	0.929
Postoperative 24 h	157 [142–182.5]	171 [162–175]	0.048^*^
PACU outcomes			
Fentanyl, μg	25 [0–50]	25 [0–50]	0.850
PCA connection time, min	20 [10–30]	20 [10–25]	0.921
Nausea	3 (7.3%)	5 (11.6%)	0.713
Vomit	1 (2.4%)	0 (0%)	0.488
Ward outcomes			
Nausea, severe/moderate/mild/none			
6 h	2/4/8/27	1/4/10/28	0.913
12 h	1/8/8/24	0/1/8/34	0.043^*^
24 h	3/6/11/21	2/9/7/25	0.574
36 h	1/6/7/27	0/1/3/39	0.040^*^
48 h	1/3/2/35	1/0/2/40	0.349
Vomiting			0.456
None	37 (90.2%)	37 (86.0%)	
1	2 (4.9%)	5 (11.6%)	
≥2	2 (4.9%)	1 (2.3%)	
Incidence of the selector-bolus line rate 0			0.134
None	23 (56.1%)	28 (65.1%)	
1	9 (22.0%)	12 (27.9%)	
≥2	9 (22.0%)	3 (7.0%)	
Total	24	18	
Reasons for the selector-bolus line rate 0			
Nausea or vomiting	20 (83.3%)	15 (83.3%)	0.269
Dizziness or headache	4 (16.7%)	2 (11.1%)	0.427
Urinary retention	0	1 (5.6%)	1.000
Antiemetics, ondansetron, mg^a^	4 [4–12]	4 [0–8]	0.049^*^
	6.1 ± 4.6	4.1 ± 3.1	
Rescue analgesics, paracetamol, g^a^	0 [0–1]	0 [0–0]	0.149
	0.76 ± 1.24	0.40 ± 0.85	
Rescue analgesics, ketorolac, mg^a^	0 [0–0]	0 [0–0]	0.543
	6.58 ± 14.25	5.58 ± 15.01	
Other adverse effects			
Headache	8 (19.5%)	6 (14.0%)	0.566
Dizziness	10 (24.4%)	9 (20.9%)	0.169
Urinary retention	0 (0%)	2 (4.7%)	0.494
Dry-mouth	5 (12.2%)	7 (16.3%)	0.183
Itchiness	0 (0%)	1 (2.3%)	0.493
Respiratory depression	0 (0%)	0 (0%)	
Length of postoperative hospital stay, day	3 [3–4]	3 [3–3]	0.266
Patients’ satisfaction score, 0–10	8 [6–10]	9 [8–10]	0.036^*^

Values are mean ± SD, median [IQR], or number of incidences (%)

Abbreviations: *PACU* post-anesthesia care unit, *QoR-40* Quality of Recovery-40 questionnaire

^*^P < 0.05

^a^Both parametric and nonparametric values are presented, although a nonparametric analysis was performed to provide an intuitive understanding of the results

In the PACU, both groups showed no intergroup differences in the use of rescue fentanyl or the incidence of nausea and vomiting (Table 2). However, in the ward, the incidence of nausea at 12 h and 36 h was higher in the 1-channel group than in the 2-channel group (*P* = 0.043 and 0.040, respectively); consequently, rescue antiemetic administration was more frequent in the 1-channel group (*P* = 0.049). The other outcomes, including the incidence of vomiting, the incidence of the selector-bolus channel with a flow rate of 0, rescue analgesics, length of postoperative hospital stay, and other adverse effects were not significantly different between the two groups. The most common reason for the selector-bolus line with a flow rate of 0 was PONV (83.3%). The overall patient satisfaction score was 8 [6–10] in the 1-channel group and 9 [8–10] in the 2-channel group, which was significantly higher (*P* = 0.036) (Table 2).

No significant differences were observed between the two groups in the NRS scores for pain at rest and while coughing in the ward during the 48-h postoperative period or in the cumulative consumption of PCA during this period in the ward (Table 3.).

**Table 3:** Numerical rating scale (0–100 scale) for pain during rest/cough and cumulative patient-controlled analgesia consumption (mL) of the continuous/selector-bolus channel in the ward

	1-channel (*n*=41)		2-channel (*n*=43)		*P* value	
NRS	Rest	Cough	Rest	Cough	Rest	Cough
					0.383	0.662
6 h	35.7 ± 22.4	52.2 ± 24.6	31.5 ± 20.2	48.4 ± 22.1		
12 h	25.6 ± 16.9	37.2 ± 19.2	22.4 ± 15.6	36.6 ± 18.6		
24 h	18.0 ± 17.8	31.7 ± 21.8	15.3 ± 14.1	28.0 ± 19.6		
36 h	16.2 ± 16.5	27.2 ± 20.5	12.6 ± 13.4	22.8 ± 17.8		
48 h	14.3 ± 15.9	23.7 ± 21.0	12.4 ± 13.9	21.7 ± 19.7		
PCA consumption	Continuous	Selector-bolus	Continuous	Selector-bolus	Continuous.	Selector-bolus
					0.639	0.535
6 h	15.2 ± 4.3	25.4 ± 5.5	14.4 ± 3.7	23.0 ± 5.8		
12 h	29.4 ± 6.4	49.0 ± 12.3	29.4 ± 5.9	46.4 ± 11.8		
24 h	55.0 ± 10.1	67.4 ± 17.7	55.2 ± 8.9	64.0 ± 16.4		
36 h	77.9 ± 9.9	78.9 ± 16.0	80.1 ± 9.1	78.7 ± 17.7		
48 h	95.0 ± 10.5	89.1 ± 16.9	95.2 ± 10.6	90.0 ± 20.7		

Values are mean ± SD

Abbreviations: *NRS* numerical rating scale, *PCA* patient-controlled analgesia

## Discussion

This randomized, double-blinded, prospective study comparing dual-channel IV-PCA and single-channel IV-PCA demonstrated comparable analgesic efficacy but a higher quality of recovery and patient satisfaction with dual-channel IV-PCA than with single-channel IV-PCA as represented by lower rates of nausea and reduced need for rescue antiemetics. These findings indicate that dual-channel IV-PCA may be a useful modality to aid recovery following TLH. To our knowledge, this study is the first to identify the usefulness of dual-channel IV-PCA.

Of the various modalities for postoperative pain management, IV-PCA is the most commonly employed method and has already become standard in recent clinical practice (Palmer and Miller 2010), and most patients prefer and are more satisfied with IV-PCA with opioids over other conventional opioid treatments (Ballantyne et al. 1993; Chumbley et al. 1999; Walder et al. 2001). Opioids, such as morphine, fentanyl, and sufentanil, are useful and potent analgesic tools for relieving moderate-to-severe postoperative pain (Oh et al. 2019). However, their usefulness is often compromised by opioid-related side effects, such as postoperative nausea and vomiting (PONV), pruritus, urinary retention, sedation, respiratory depression, and confusion (Grass 2005; Oh et al. 2019).

Among these opioid-related side effects, PONV is the most common and bothersome; therefore, it frequently complicates recovery from surgery. In our study, the interruption of main drug line infusion (setting the selector-bolus line with a flow rate of 0) mainly came from PONV (83.3%) than other side effects such as dizziness, headache, or urinary retention. Even mild PONV can increase the length of stay in the hospital, decrease patient satisfaction, and lead to increased healthcare costs (Hill et al. 2000). Avoiding PONV is more important than avoiding postoperative pain when regarding patients’ preferences for postoperative anesthesia outcomes (Macario et al. 1999). Therefore, PONV can be considered the main factor underlying patient satisfaction and quality of recovery. The risk factors for PONV include female sex, a history of motion sickness or PONV, nonsmoking status, and postoperative opioid use (Gan et al. 2014). Our study population might be susceptible to PONV because they were female patients who were receiving postoperative opioids. Hence, the dual-channel PCA closely affected patient recovery and satisfaction with a lower incidence of PONV and lower rescue antiemetic use by eliminating the period of suspended adjuvant drug infusion, although the main drug line infusion is stopped by setting the PCA device. The dual-channel PCA not only delivered the antiemetic agents or adjuvant analgesics in a situation where the main drug infusion was suspended, but also lowered the need for additional rescue intervention. Frequent rescue intervention is not consistent with the intended purpose of the PCA, which maintains a target analgesic concentration based on patient demand, and this may increase patient discomfort. In our study, the PONV incidence was frequent at 12 h and 36 h postoperatively but not 6 h, 24 h, and 48 h postoperatively. This might reflect the failed management of PONV during the suspended periods (setting the selector-bolus line with a flow rate of 0). Additional antiemetics might relieve the symptoms, but the symptoms could be re-activated by resuspension.

It was interesting that by simply dividing the same regimen of drugs into different channels, patient satisfaction, and recovery were improved with the same level of drug consumption. In this regard, the main advantage of the dual-channel PCA is that the concentration of adjuvant analgesic agents and antiemetic agents can be maintained constantly, even if the infusion of the opioid is stopped. On the contrary, when the conventional single PCA is suspended due to opioid-related side effects, such as PONV, the antiemetic agents are also suspended, so rescue agents may be needed and the concentration of opioids may fall under the minimum effective analgesic concentration, consequently leading to a rise in the pain level (Grass 2005).

PCA has various administration modes. The two most commonly used modes are demand dosing (a fixed dose is self-administered intermittently) and continuous infusion plus demand dosing (a constant-rate background infusion is supplemented by patient demand dosing). Almost all modern PCA devices provide both modes (Grass 2005). Unlike the modes mentioned above, the mode used in our study was variable-rate infusion (2 or 4 mL/h; 2 mL/h fixed rate of adjuvants in the continuous channel plus 0 or 2 mL/h of opioids in the selector-bolus channel) plus demand dosing of opioids. Although the selector-bolus channel has a range of 0, 1, and 2 mL/h, which is adjustable using a dial, only 0 or 2 mL/h were used in both groups in our study. The reason is that the typical single-channel PCA device has a constant-rate continuous infusion mode plus demand dosing, and the constant-rate continuous infusion is suspended at a rate of 0 when the infusion is stopped. This is reflected in the 1-channel group as the control group. Therefore, utilizing various flow rate options only in the experimental (2-channel) group might be over-beneficial to the 2-channel group and may unblind the groups.

There has been an issue with using basal infusion in the opioid-based IV-PCA. Although the basal infusion may improve pain management (Sinatra et al. 1989), it simultaneously increases the incidence of opioid-related side effects including opioid-induced ventilatory impairment and PONV (Jung et al. 2020; Parker et al. 1991). With morphine, the routine use of basal infusion is not recommended (Chou et al. 2016). In contrast to the longer-acting opioids such as morphine, with sufentanil, which has a short half-life of clearance and relatively higher therapeutic index, basal infusion may be required; otherwise, patients will require frequent boluses (Oh et al. 2019). With fentanyl, which has different pharmacokinetic properties, the evidence of the risks and benefits of basal infusion has been insufficient. A recent study reported that basal infusion of fentanyl-based IV-PCA significantly increased the consumption of fentanyl and the occurrence of opioid-related side effects in postsurgical patients (Jung et al. 2020). In our study, the basal infusion was used at the start, but setting the rate 0 of the selector-bolus line, which means the basal infusion stopped but the bolus functions, reflects the condition of eliminating basal infusion in the fentanyl-based IV-PCA. Setting the rate 0 of the selector-bolus line in the 2-channeled PCA, which means no opioid basal infusion but the basal infusion of adjuvants, might facilitate the patient management.

The usefulness of this device has just begun to be explored; therefore, various options and modes of this device are expected to be applied in various situations. One possible option is to apply drugs according to the procedure-specific analysis of characteristics of postoperative pain. In a patient undergoing gynecological laparoscopic surgery who is expected to suffer from incisional, abdominal visceral, and shoulder pain, two different opioids acting on different receptors can be applied via different channels. For example, full agonists of the μ-opioid receptor, such as morphine, fentanyl, or sufentanil, can be used for somatic pain, and κ-opioid receptor agonists such as oxycodone can be used for visceral pain, considering the pivotal role of κ-opioid receptors in visceral pain attenuation (Han et al. 2018; Nielsen et al. 2007). Another option is volume and concentration adjustment in each channel. Considering that the intensity of postoperative pain is gradually decreased in general (Chitnis et al. 2020), continuous infusion at a constant rate may be inappropriate during the entire first 48 h postoperatively. Therefore, a low concentration of opioids can be administered via the continuous channel with a full volume of 100 mL to maintain the minimum effective analgesic concentration, and other the selector-bolus channel is filled with relatively higher concertation opioids with a half volume of 50 mL for acute phase pain control during the first 24 h postoperatively. This method may also prevent periods of more than the optimal plasma drug concentration resulting in opioid-induced ventilatory impairment (Bowen et al. 2020; Grass 2005) in the late phase after surgery. This disposable PCA can be used in a programmed mode without using microprocessor-driven PCA devices.

With the increasing interest in enhanced recovery after surgery, perioperative multimodal analgesia using combinations of analgesics that act on different sites and pathways in an additive or synergistic manner to achieve pain relief has been highlighted (Beverly et al. 2017). However, mixing different drugs in the same syringe or pump is not recommended because of the incompatibility of mixtures including precipitation, ionic reactions, and evolution of gas (Murney 2008). In addition, as individual opioid requirements vary widely, the adjuvant drug delivered consumption will also vary. Therefore, the practice of combining drugs in the same pump could yield an inadequate effect of the adjuvant drug in some patients and an excessive effect in others (Macintyre 2001). Therefore, utilizing the dual-channel PCA pump facilitates the combination of analgesics with different mechanisms and other adjuvant treatments. Patient-centered personalized postoperative pain management will be expected to be widely applied using this dual-channel PCA device.

This study has several limitations. First, even though the importance of avoiding different drugs mixture in the same pump was addressed, drugs were mixed in both groups (3 drugs in 1 channel group and 2 drugs in 2 channel group). According to our researchers’ in vitro laboratory study (Kim et al. 2021), in the mixture of fentanyl, ketorolac, and ondansetron, safety issues, such as precipitation and incubation, did not happen, but the decrease in the concentration of ketorolac might happen at 24 h and 48 h. This might affect our result. Second, the practices applied in this study were somewhat conventional and specified in the patients undergoing TLH in the Korean insurance and reimbursement system (relatively low charge). Therefore, it may be difficult to generalize our regimens. However, it is thought that this did not affect the effect of dual-channel PCA itself.

## Conclusions

The 2-channel PCA showed better patient satisfaction with higher QoR-40 during the recovery compared with the 1-channel PCA. Better satisfaction was associated with lower rates of nausea and reduced need for rescue antiemetics by maintaining the infusion of adjuvant analgesic agents and antiemetic agents constantly by utilizing dual channels. Our study explored the benefit of dual channel PCA over conventional single channel use, but the clinical benefits of applying various modes of this device should be further studied.

## Data Availability

The datasets used and analyzed during the current study are available from the corresponding author on reasonable request

## Abbreviations

PCA: Patient-controlled analgesia
QoR-40: Quality of Recovery-40 questionnaire
TLH: Total laparoscopic hysterectomy
ASA: American Society of Anesthesiologists
PACU: Post-anesthesia care unit
VPS: Verbal pain score
NRS: Numeric rating scale
SD: Standard deviation
IQR: Interquartile range
PONV: Postoperative nausea and vomiting
